# Polycyclic aromatic hydrocarbons in the coastal sea water, the surface sediment and Mudskipper *Boleophthalmus dussumieri* from coastal areas of the Persian Gulf: source investigation, composition pattern and spatial distribution

**DOI:** 10.1186/2052-336X-12-59

**Published:** 2014-03-10

**Authors:** Mahmood Sinaei, Ali Mashinchian

**Affiliations:** 1 Department of Fisheries, Chabahar Branch, Islamic Azad University, Chabahar, Iran; 2 Department of Marine Chemistry, Graduate School of Marine Science and Technology, Science and Research Branch, Islamic Azad University, Tehran, Iran

**Keywords:** Coastal waters, Sediments, Biota, Liver, Gill, *Boleophthalmus dussumieri*

## Abstract

**Background:**

Persian Gulf is an exposed and stressed area as a result of oil pollution and other fossil fuels containing PAHs. The susceptibility of using mudskippers to monitor marine pollution, like PAHs, points to the fact that mudskippers are able to accumulate and record the PAHs presented in the coastal environments.

**Methods:**

Polycyclic aromatic hydrocarbons (PAHs) were examined in the coastal waters, the sediments and biota (i.e., *Boleophthalmus dussumieri*) along the coast of the Persian Gulf. PAHs concentrations were measured with HPLC method.

**Results:**

Total PAH concentrations in the sea water, the sediments, the liver and the gill tissues ranged between 0.80-18.34 μg/l, 113.50-3384.34 ng g^-1^ (d w), 3.99-46.64 ng g^-1^ (d w) and 3.11-17.76 ng g^-1^ (d w), respectively. PAHs distribution patterns in the sediment and the liver tissue samples were dominated by three-and four-ring structures whereas two-and three-rings were dominated in the water and the gill.

**Conclusions:**

This finding revealed a negative eco-risk effects occasionally occur in this area. The higher presence of low condensate ring structures reflected a predominant origin of petrogenic and some cases of pyrolitic sources.

## Background

Marine environments have become an easily reasonable exposure site for pollutants such as polycyclic aromatic hydrocarbon (PAH) compounds. PAHs are a group of environmental contaminants formed during the incomplete combustion of organic materials such as coal, fossil xfuel, and wood, as well as from forest fires, volcanic activity and petroleum seeps [1-5]. PAHs are well known environmental pollutants and are included in priority pollutant list of the European Union and US Environmental Protection Agency (EPA) due to their mutagenic and carcinogenic properties [6,7].

Source determination of PAH in the marine environment seems to be difficult, because they are produced by three major processes: Pyrogenic PAH, Petrogenic PAH and Diagenic PAH. Pyrogenic PAHs result from an incomplete but high temperature and short-duration combustions of organic matters including fossil fuels and biomass [3,8]. Petrogenic PAHs are formed by biogenic processes at a relatively low temperature over geologic time scale, leading to the formation of petroleum and other fossil fuels containing PAHs [3,9]. Diagenic PAHs refer to PAHs from biogenic precursors, like plant terpenes [10,11]. The first two types of sources may be both natural and anthropogenic sources. Petrogenic PAH dominates oil-polluted samples, while Pyrogenic PAH tends to dominate the samples from industrial areas. The toxicity, environmental persistence, bioaccumulation, and trophic transfer of PAHs in aquatic ecosystem are vital to assess the ecological risk of contaminants [12-14].

All kinds of Fish definitely respond to pollutants by altering/adapting their metabolic functions. Hence, Fish have been often used as appropriate bioindicators of the chemical contaminants [15-17]. Mudskippers (Gobiidae: Oxudercinae) live in intertidal mudflats and in mangrove ecosystem [18]. They spend much of their life buried, being in close contact with the sediments [12]. The susceptibility of using mudskippers to monitor marine pollution, like PAHs, points to the fact that mudskippers are able to accumulate and record the PAHs presented in the coastal environments. Therefore, the analysis of mudskipper tissues may give an indication of the bioavailable portion of environmental PAH contamination [12].

The Persian Gulf is considered as one of the most polluted ecosystems in the world. It is an exposed and stressed area as a result of oil pollution [19]. The environmental condition of the Persian Gulf became more critical because of the war related activities [20]. A large number of studies have addressed PAHs in the Persian Gulf, mainly to determine concentration, composition and degradation [15,19,21-29]. In line with these trends of research, this study was conducted: the objective of the present paper was to investigate the levels of PAHs in *B. dussumieri*, water and sediment samples collected from this area.

## Materials and methods

### Study species

Mudskippers (i.e., *B. dussumieri*) were selected as the main organism for this research. A Mudskipper has a preferential uptake of PAHs from the sediments due to its benthic characteristics. This is considered important because the most carcinogenic PAHs are not usually present in the dissolved phase in a marine environment. These compounds are bound to the sediments or the suspended matters. Accordingly, Mudskippers (*B. dussumieri*) have been used in this research as a sentinel organism for the common chemical analysis in the coastal areas of the Persian Gulf.

### Study sites

**Figure 1:**
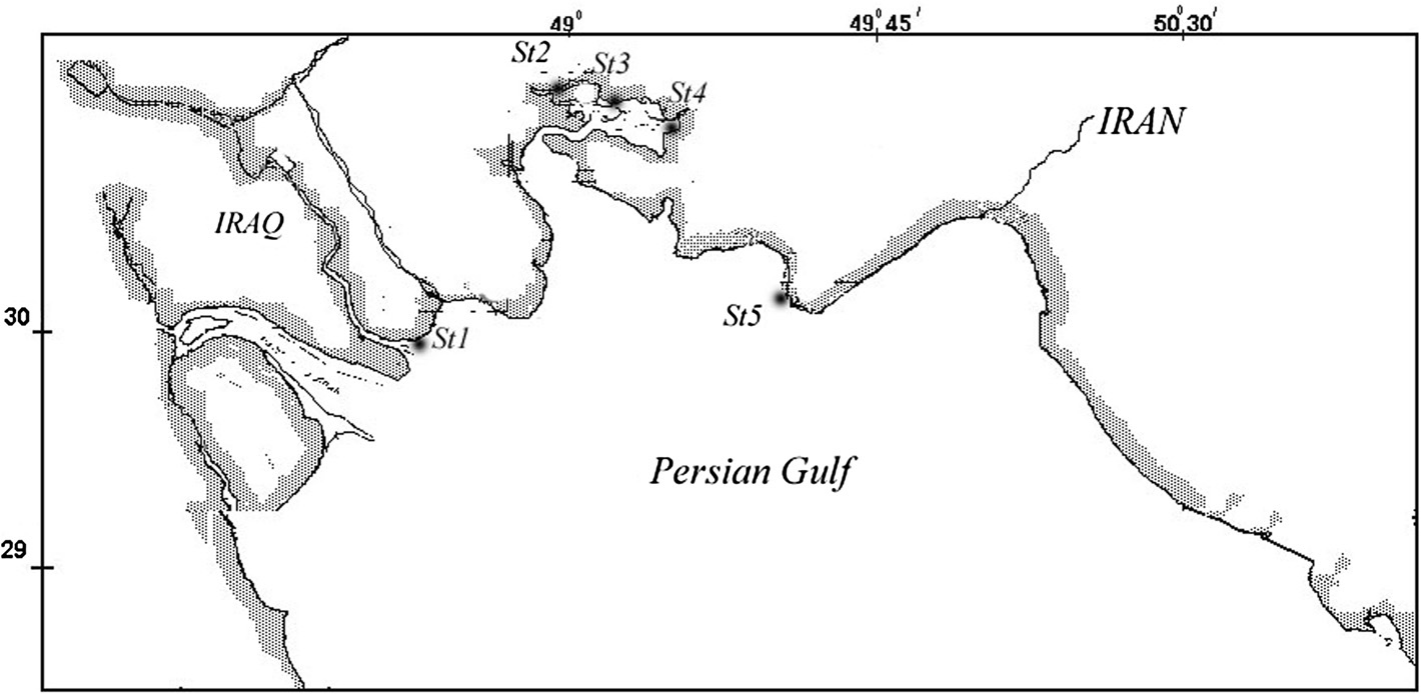
Location of all sampling sites.

Five different stations were chosen along the north western coast of the Persian Gulf (see Table 1 and Figure 1). Most of the locations were near the mouths of discharges of commercial and domestic waste. Furthermore, they were selected along a doubtful pollution gradient based on the earlier information available in literature about the local contaminant levels [30].

**Table 1:** Background information on the sampling stations in the North West of Persian gulf

**Sampling sites**	**Coordinates**		**Description**
	**Latitude**	**Longitude**	
**Arvand(St1)**	48º 40′58″E	29º54′35″N	Abadan refineries
**Jafari(St2)**	49º06′52″E	30º26′52″N	Special economic and petroleum zone, urban and industrial dump
**Zangi(St3)**	49º03′52″E	30º28′52″N	landing port for vessels, petroleum industries
**Samayeli(St4)**	49º10″51″E	30º27″45″N	landing port for fishing vessels, petroleum saving tank
**Bahrakan(St5)**	49º50′54′ E	30º08′14″N	Reference site

St: Station.

### Sampling and preparation for chemical analysis

The sediments, the coastal water and the biota (i.e., B. dussumieri) were collected in October 2011. The mudskippers (n = 30) were collected from each of the five sampling sites by using hand nets. For the transportation of the fish to the laboratory, they were stored inside the aquaria with a volume of 60 L (semi-static to be aerated under ambient conditions: a water temperature of 25.2 ± 0.7°C, a salinity of 38 g/l).

Immediately after transportation of the fish to the laboratory, mudskippers were anesthetized by pouring dry extracts of pink cloves into water column. Subsequently, the fish were removed and were all measured in length and weight: a mean length of 17 ± 0.1 cm and a mean weight of 16 ± 0.2 g. The fish were immediately dissected and the liver was quickly removed at the ice. The Liver tissues were wrapped in the foils and were stored at -20°C for the following PAHs pending analyses.

Superficial sediment samples (top 8 cm) were taken from five different locations on each of the five study sites. The sediment samples were placed in a Teflon-lined amber vial on an ice pack until it could be stored at -20°C to be ready for the following PAHs analysis. These steps were taken following the standards of ICES ACME Report 1997 [31].

Coastal sea water was collected in acid-washed amber glass bottles fitted with Teflon screw caps from three different locations at each five study sites. To remove suspended matter, samples were filtered through glass fiber filters (Whatman GF/C). Filtered samples were stored in pre-cleaned (with distilled water) glass bottles at 4°C prior to the PAHs analysis.

### Extraction and purification of PAHs in tissues

In order to extract and purify PAHs, the same method proposed earlier by Perugini et al., in 2007 was followed [32]. This determination was carried out in composite pools of tissues dissected from 30 fish (five samples, each constituted by tissues of six specimens). After being thawed, the assay sample (2 g dw) was inserted into a l00mL round-bottomed flask with a 10 mL of 1 M KOH in an ethanolic solution. The mixture was placed in a reflex system in a temperature of 80°C for 3 hours. The liquid phases were transferred to a separation funnel and were extracted with a 10 mL of cyclohexane. They were then shaked rigorously for 30 minutes. The cyclohexane KOH phase was drained and discarded. Next, the liquid phases were rinsed with 10 mL cyclohexane once more. The samples were allowed to pass through the anhydrous sodium sulphate column. After that, the organic phase was concentrated in a rotary evaporator (Model Buchi B-490) to a volume of 5 mL under a reduced pressure. The samples passed through a column filled with florisil and concentrated in rotary evaporator (30°C, 19–21 kPa) to volume of 1 mL. Finally, the extracts were evaporated with a gentle stream of nitrogen at room temperature and were then reconstituted in 1 mL of acetonitrile.

### Extraction and purification of PAHs in sediment

For the extraction and purification of PAHs the method proposed by Moopam in 1999 was adopted [33]; however, this was done by implementing some slight modifications based on the context of the present research. PAHs in the sediments (N = 25) obtained from five different locations on each of the five study sites were measured. Sediment samples (each 200grams) were freeze dried (freeze-drier Model: OPR-FDB-5503, Korea) at -40ºC to a constant weight. Subsequently, a 10 g of each sample were mixed with a 250 mL of n-hexane and dichloromethane mixture (1:1 ratio). Following that, the mixture was placed for 8 hours in Soxhelet. The combined extracts were concentrated to about 1 mL by vacuum rotary evaporation. To eliminate sulphur and the related compounds, a 3 g of active copper was added to the mixture and was then allowed to pass through a folded filter paper (Whatman GF/C, 24micron) for 24 hours. The mixture was concentrated in a rotary (Buchi B-490) to a volume of 5 mL. The Samples were passed through a column which contained a 10 g of silica gel in n-hexane, 1-2 g of anhydrous sodium sulphate, and a 10 mL of alumina. Then, a 30 mL of n-hexane and dichloromethane mixture (9:1 ratio) was added to the column. The samples were concentrated once more in a rotary evaporator to a volume of 5 mL. After that, the concentrated extract was dried under the nitrogen steam (N-E VAP 112, USA). The residue was finally dissolved in a 1 mL of acetonitrile.

### Extraction and purification of PAHs in water

Extraction and purification of PAHs in water was conducted as the following method. The experimental procedure is based on the USEPA Method 550.1 of Bashe and Baker in 1990 [34]. Solid phase partitioning was carried out by processing 500 ml of the sample through a 25 mm C18 Empore TM extraction disk mounted in a side arm vacuum flask for approximately 30 min. The disks were dried and desorption was performed by eluting with 4 × 5 ml of acetonitrile. Samples were concentrated to 0.5 ml under a gentle stream of nitrogen. PAHs in the particulate fraction were isolated from the glass fiber filters with 4 _ 5 ml of acetonitrile by ultrasonication during 30 min. Samples were then centrifuged and concentrated to 0.5 ml under nitrogen steam.

### Chemical analysis

The sixteen most toxic PAHs introduced by the Environmental Protection Agency (EPA) were assessed in the coastal waters, the sediments and the biota (mudskipper: B. dussumieri). The abbreviations used for the investigated PAHs are: naphthalene: N; acenaphthylene: AC; acenaphthene: ACE; Fluorene: F; phenanthrene: Ph; anthracene: A; fluoranthene: Fl; pyrene: Py; benz(a)anthracene: B(a)A; chrysene: Ch; benzo(b)fluoranthene: B(b)Fl; benzo(k)fluoranthene: B(k)Fl; benzo(a)pyrene: B(a)Py; indeno(1,2,3-c,d)pyrene: IPy; dibenz(a,h)anthracene: DB(ah)A; benzo(ghi)perylene: B(ghi)Pe. Hewlett-Packard 1100 HPLC equipped with an Agilent-1100 fluorescence detector was used. Injection volume was 10 μL. The initial mobile phase was a 60% of acetonitrile and a 40% of HPLC water for 40 minutes with a flow rate of 0.7 mL min^-1^, which was then gradiently changed to 100% acetonitrile.

### Gross morphometric indices

Gross morphometric indices were computed for each individual Mudskipper consenting to the following formulas:

$$\begin{array}{c} \mathrm{Condition}\;\mathrm{factor}\;\left(\mathrm{CF}\right):\left(\mathrm{WT}/3\right)\times 100\\ \mathrm{Hepatosomatic}\;\mathrm{index}\;\left(\mathrm{HSI}\right):\left(\mathrm{WH}/\mathrm{WT}\right)\times 100 \end{array}$$

Where WT: total wet weight (g); L: total length (cm); WH: liver weight (g).

### Quality control

Replicate samples, certified reference materials IAEA-417 and procedural blanks were used as quality control procedures. All the obtained values for PAHs in CRM were in the reported range. Reproducibility and recovery were high (85–110%) with relative standard deviation (RSD) 4–11%. To measure the quality control, the procedural blanks were periodically analyzed for each batch of 10 samples. Quantitative analysis was conducted on a five-point linear calibration of PAH solution, obtained by dilution of the certified standard mixture of 16-PAH (kit 610-N-Supelco4-7351). Blank samples were processed together with samples, and limits of detection (LODs) were estimated as the average signal of the blanks plus five times the standard deviation of the signal of the blanks. LODs for biota, sediment and water measurements ranged from 0.18 to 0.4 ng g^_1^ dw and 0.5–1.4 ng g^_1^ dw, 0.1-0.12 μg/l respectively. Satisfactory linearity was obtained with values of the correlation coefficient R above 0.99.

### Statistical analyses

Statistical analysis was done using the SPSS software (version 19). The data were tested to check for the normality using the Kolmogorov–Smirnov test and indicated a normal distribution. To assess changes in PAHs levels at different sampling sites, One way analysis of variance (ANOVA), followed by Tukey’s multiple range test for mean comparisons was applied (p < 0.05).

## Results

### PAH pollution of the sediments

**Figure 2:**
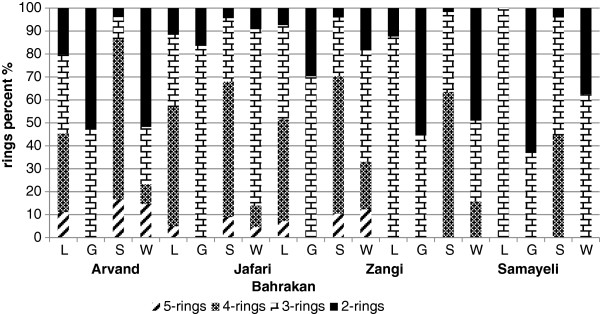
Composition pattern of PAHs in sampling sites.

Poly aromatic hydrocarbons contents determined in the sediment from five sampling sites in the Persian Gulf as well as the results of the related statistical analyses are displayed in Table 2. Among 16 PAHs, levels of B(a)A represented the highest concentration among all sampling sites. The highest concentration of total PAHs (Σ16PAH) was measured at Jafari followed by Zangi > Arvand > Samayeli > and Bahrakan. For total PAHs levels (Σ16PAH), significant variations in all of the sampling sites were also found (p < 0.05). The composition patterns of PAHs in all the sites were dominated by the presence of four-ring PAH except for the PAH in Bahrakan where three-ring structures followed by four-ring ones prevailed (Figure 2).

**Table 2:** **Mean and standard deviation + 16PAHspercent per T-PAHs values of sediment (ng g**^**-1 **^**dry weight) and water (μg/l) collected from different sites**

**PAHs**	**Samples**	**Arvand**	**Jafari**	**Zangi**	**Samayeli**	**Bahrakan**
N	S	nd	nd	nd	nd	nd
	W	nd	nd	nd	nd	nd
AC	S	13.297 ± 1.451^a^	132.112 ± 9.90^b^	74.903 ± 6.015^c^	1.979 ± 0.461^d^	nd
	W	1.584 ± 0.331^a^	1.121 ± 0.155^a^	0.810 ± 0.110^a^	2.634 ± 0.522^b^	nd
ACE	S	nd	nd	nd	nd	nd
	W	nd	nd	nd	nd	nd
F	S	44.005 ± 1.590^a^	17.353 ± 1.480^b^	3.323 ± .792^c^	4.319 ± .690^c^	4.698 ± .690^c^
	W	1.123 ± 0.223^a^	0.566 ± 0.026^b^	0.286 ± 0.065^c^	0.341 ± 0.031^c^	0.300 ± 0.026^c^
Ph	S	20.989 ± 2.769^a^	632.682 ± 27.72^b^	33.429 ± 2.690^a^	36.861 ± 3.003^a^	15.438 ± 1.64^a^
	W	0.406 ± 0.028^a^	11.104 ± 1.391^b^	0.449 ± 0.051^a^	0.455 ± 0.040^a^	0.373 ± 0.014^a^
A	S	nd	nd	nd	nd	nd
	W	nd	nd	nd	nd	nd
Fl	S	5.164 ± 0.237^a^	178.975 ± 9.38^b^	29.546 ± 3.001^c^	17.445 ± 2.432^d^	14.289 ± 1.74^d^
	W	0.734 ± 0.091^a^	2.818 ± 0.632^b^	2.162 ± 0.467^bc^	1.472 ± 0.371^ac^	nd
Py	S	114.260 ± 4.645^a^	126.051 ± 7.979^a^	426.015 ± 14.55^b^	71.298 ± 6.074^c^	28.476 ± 3.46^d^
	W	0.167 ± 0.080^de^	0.174 ± 0.015^de^	0.348 ± 0.054^c^	0.208 ± 0.023^d^	0.125 ± 0.040^e^
B(a)A	S	623.201 ± 18.16^a^	1132.562 ± 59.923^b^	649.151 ± 20.06^a^	188.623 ± 8.388^cd^	33.220 ± 3379^d^
	W	0.453 ± 0.015^a^	0.565 ± 0.083^a^	0.460 ± 0.064^a^	0.308 ± 0.072^b^	nd
Ch	S	330.579 ± 16.05^a^	760.706 ± 7.28^b^	307.695 ± 14.09^a^	40.299 ± 3.245^cd^	17.379 ± 1.491^d^
	W	nd	1.150 ± 0.190^a^	0.791 ± 0.089^a^	0.645 ± 0.121^a^	nd
B(b)Fl	S	26.360 ± 3.203^a^	59.813 ± 4.120^b^	111.277 ± 7.901^c^	Nd	nd
	W	nd	nd	nd	nd	nd
B(k)Fl	S	27.968 ± 3.721^a^	41.532 ± 4.198^b^	72.833 ± 4.854^c^	nd	nd
	W	nd	nd	nd	nd	nd
B(a)Py	S	240.500 ± 12.494^a^	302.557 ± 14.891^b^	199.636 ± 7.206^c^	nd	nd
	W	0.763 ± 0.025^a^	0.839 ± 0.033^a^	0.741 ± 0.109^a^	nd	nd
IPy	S	nd	nd	nd	nd	nd
	W	nd	nd	nd	nd	nd
DB(ah)A	S	nd	nd	nd	nd	nd
	W	nd	nd	nd	nd	nd
B(ghi)Pe	S	nd	nd	nd	nd	nd
	W	nd	nd	nd	nd	nd
Ʃ PAH	S	1446.323 ± 200.950*	3384.34 ± 376.946*	1907.81 ± 210.486*	360.82 ± 65.028	113.50 ± 10.334*
	W	5. 23 ± 0.479^a^	18.34 ± 3.648^b^	6.12 ± 0.603^a^	6.06 ± 0.887^a^	0.80 ± 0.127^d^

Values followed by the same letters (a, b, c, d) are not statistically different between the 16PAHs in each row (P > 0.05).

*Indicates significant difference between the Ʃ PAH in each row (P > 0.05); nd = not detected; S = sediment; W = water.

### PAH pollution of the water

The results of statistical analysis of 16 PAH levels in the coastal water samples are presented in Table 2. For the total PAHs levels (Σ16PAH), the highest concentrations were detected at Jafari followed by Zangi > Samayeli > Arvand > and Bahrakan. Significant differences were found between Jafari and Bahrakan and with other sites (p < 0.05); however no significant difference was revealed among Arvand, Zangi and Samayeli sampling sites (p > 0.05). The composition patterns of PAHs was prevailed by two-ring structures at Arvand and Samayeli, whiles three-ring structures dominated in three other sites followed by those with two-ring (Figure 2).

### PAHs concentrations in liver tissues

The results of the determination of 16 PAH accumulated in liver tissue of mudskipper together with the results of the statistical analysis are shown in Table 3. The levels of PAHs in the liver from the five sites showed that only nine PAHs could be identified at detectable levels. For the total PAHs levels (Σ16PAH) in the liver, the highest concentration was detected at Jafari followed by Zangi > Arvand > Samayeli > and Bahrakan. When comparing concentration of total PAHs among sites, there were only significant difference between Jafari and Bahrakan (p < 0.05). However, there was no significant difference between the other sites compared two by two (p > 0.05). Samayeli and Bahrakan sites were prevailed with three-ring structures. Moreover, a three -and four-ring PAH structures prevailed in Arvand section (Figure 2).

**Table 3:** **Mean and standard deviation + 16PAHspercent per T-PAHs values of liver and gill tissues (ng g**^**-1 **^**dry weight) collected from different sites**

**PAHs**	**Tissues**	**Arvand**	**Jafari**	**Zangi**	**Samayeli**	**Bahrakan**
N	L	nd	nd	nd	nd	nd
	G	nd	nd	nd	nd	nd
AC	L	3.532 ± 0.49^a^	4.381 ± 0.54^a^	1. 829 ± 0.177^b^	2.233 ± 0.55^b^	nd
	G	1.956 ± 0.580^bc^	1.934 ± 0.694^bc^	1.432 ± 0.382^c^	3.009 ± 0.984^d^	nd
ACE	L	nd	nd	nd	nd	nd
	G	nd	nd	nd	nd	nd
F	L	2.471 ± 0.55^a^	1.051 ± 0.132^b^	0.558 ± 0.083^b^	0.660 ± 0.095^c^	nd
	G	2.137 ± 0.570^a^	0.993 ± 0.187^b^	0.980 ± 0.191^cb^	1.450 ± 0.344^ab^	1.963 ± 0.621^a^
Ph	L	3.576 ± 0.676^a^	9.265 ± 0.826^b^	2.619 ± 0.449^a^	3.073 ± 0.625^a^	1.053 ± 0.330^e^
	G	1.218 ± 0.287^ce^	11.388 ± 1.630^b^	1.761 ± 0.501^c^	1.342 ± 0.430^ce^	0.177 ± 0.048^e^
A	L	nd	nd	nd	nd	nd
	G	nd	nd	nd	nd	nd
Fl	L	1.019 ± 0.435^a^	3.222 ± 0.763^b^	1.036 ± 0.431^a^	4.119 ± 0.337^db^	nd
	G	1.623 ± 0.316^a^	3.118 ± 0.402^b^	3.190 ± 0.847^b^	1.562 ± 0.388a	nd
Py	L	5.634 ± 0.830^a^	2.168 ± 0.468^b^	9.586 ± 1.030^c^	13.296 ± 0.976^b^	2.932 ± 0.792^b^
	G	0.860 ± 0.140^a^	0.326 ± 0.0.40^b^	0.736 ± 0.180^a^	0.665 ± 0.090^a^	0.973 ± 0.126^a^
B(a)A	L	5.275 ± 1.110^a^	14.460 ± 2.931^b^	7.153 ± 1.451^c^	nd	nd
	G	nd	nd	nd	nd	nd
Ch	L	4. 110 ± 0.627^a^	9.828 ± 1.270^b^	4.097 ± 0.897^a^	nd	nd
	G	nd	nd	nd	nd	nd
B(b)Fl	L	0.750 ± 0.183^a^	nd	3.036 ± 0.847^b^	nd	nd
	G	nd	nd	nd	nd	nd
B(k)Fl	L	nd	nd	nd	nd	nd
	G	nd	nd	nd	nd	nd
B(a)Py	L	3.307 ± 0.827^a^	2.263 ± 0.687^b^	2.303 ± 0.524^b^	nd	nd
	G	nd	nd	nd	nd	nd
IPy	L	nd	nd	nd	nd	nd
	G	nd	nd	nd	nd	nd
DB(ah)A	L	nd	nd	nd	nd	nd
	G	nd	nd	nd	nd	nd
B(ghi)Pe	L	nd	nd	nd	nd	nd
	G	nd	nd	nd	nd	nd
**Ʃ PAH**	L	**29.67 ± 1.678**^**a**^	**46.64 ± 4.782**^**b**^	**32.22 ± 2.971**^**a**^	**23.38 ± 4.981**^**a**^	**3.99 ± 1.328**^**d**^
	**G**	**7.79 ± 0.524**^**a**^	**17.76 ± 4.504**^**b**^	**8.10 ± 0.963**^**a**^	**8.03 ± 0.858**^**a**^	**3.11 ± 0.894**^**c**^

Values followed by the same letters (a, b, c, d) are not statistically different between the 16PAHs in each row (P > 0.05) nd = not detected; L = Liver; G = Gill.

### PAHs concentrations in gill tissues

**Figure 3:**
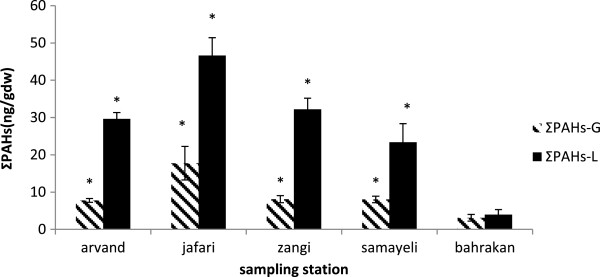
PAHs ± S.D in liver and gill tissues of mudskipper.

The results of the determination of 16 PAH accumulated in gill tissue of mudskipper together with the results of the statistical analysis are shown in Table 3. As clearly observable form the table, only five PAHs (Py, Fl, Ph, F, and AC) can be identified at the detectable levels, Nevertheless, among the five PAHs pollutant detected in the gill samples, only three ones (Py, Fl, Ph) were found at Bahrakan site. The highest concentrations of the total PAHs levels (Σ16PAH) in the gill were found at Jafari site followed by Zangi > Samayeli > Arvand > and Bahrakan. As Figure 3 displayed, PAHs were accumulated more significantly in the liver than in the gill tissues in all stations except at Bahrakan (p < 0.05). The composition pattern of PAHs in the gill tissues was prevailed by two-ring structures at Arvand, Samayeli and Bahrakan, On the other hand PAHs were dominated with three-ring structures at two other sites, that is, Jafari and Zangi (Figure 2).

### Biological indices

**Figure 4:**
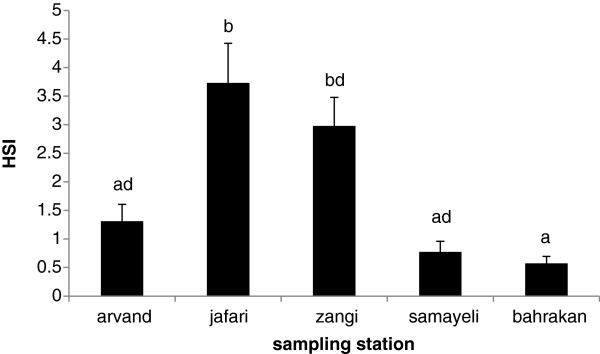
HSI ± S.D values in different stations.

**Figure 5:**
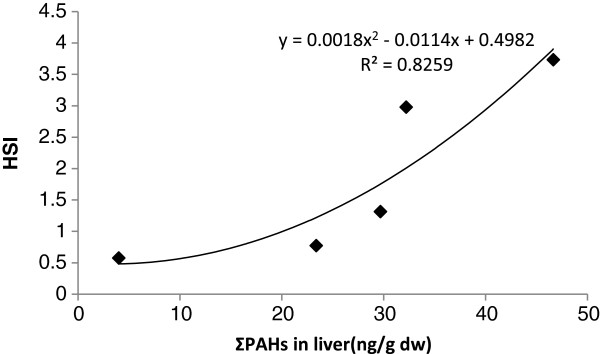
Correlation between HSI and PAHs in liver.

**Figure 6:**
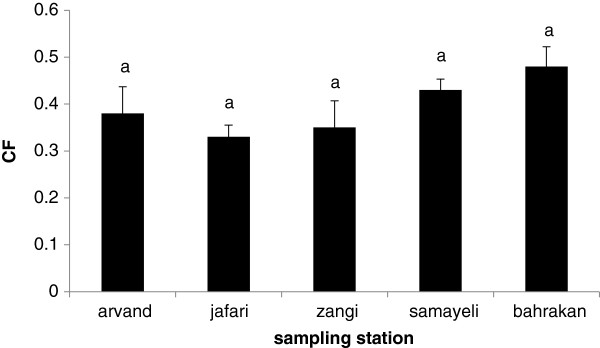
CF ± S.D values in different stations.

The HSI value results and the related statistical analysis are illustrated in Figure 4. A strong correlation (r =0.90) was found between HSI and total PAHs (Σ16PAH) in the liver samples (Figure 5). In this study, CFs were found in the fish collected from all five sites. As Figure 6 showed, the CFs of the fish living in the stations were not significantly different (p > 0.05).

### Origin of the contaminants

To characterize origin of PAH contamination more appropriately, according to the processes from which they were generated, molecular indices were developed based on the thermodynamic stability of various isomeric compounds. However, because of the complexity of the parameters governing PAH distributions in the environment, simultaneous consideration of these molecular indices is necessary to be able to distinguish PAHs originating from various sources [35-38] Accordingly, three PAH ratios (phenanthrene/anthracene, fluoranthene/pyrene and chrysene/benzo(a)anthracene) were selected, These ratios were also suggested by other researchers [12,35,36] for the identification of pyrolytic or petrogenic origin of PAHs. The former refers to combustion emissions, and the latter addresses oil petroleum spillages, industrial effluents, and urban discharges. However, we could not use all of the ratios because levels of anthracene concentrations in some stations were below the detection limit of the instrument applied.

In the case of the sediment and the liver samples, Fluoranthene/Pyrene ratios were found to be lower than 1 except for those at Jafari site. The value of Fluoranthene/Pyrene ratios were more than 1 for the water and the gill samples except at Bahrakan site. Chrysene/benzo(a)anthracene ratios were more than 1 for the sediment and the liver samples at Arvand, Jafari and Zangi sites, but not the same result was found at two other sites. Chrysene/benzo(a)anthracene ratios for the water samples were found to be higher than 1 at Arvand, Jafari; nevertheless, it was lower than 1 at Zangi sites.

## Discussion

Persian Gulf is considered as one of the most pollutant marine ecosystems in the world. Almost, two-thirds of the world’s proven oil reserves are located in the Persian Gulf [39]. This region has undergone considerable development, urbanization and industrialization; port areas have become the major sources of pollution in marine environments.

Khuzestan coast in the north western of the Persian Gulf was selected, since the problems associated with oil pollution have been perceived to be of crucial importance. Ports, refineries and other petroleum industrial wastes have had significant influence on this area, suggesting that the coastal animals are probably exposed to PAHs pollution. Mudskipper (*B. dussumieri*) was selected since they were abundant in the area, and they had also the potentiality to function as regional bioindicator.

The total amount of 16 PAH concentration did not exceed the NOAA sediment quality guideline value for the effects range low (ERL) of 4022 ng/g dry weight at five sampling sites. The composition patterns of the PAHs in the sediment samples were dominated by three-and four-ring structures. Different orders of the PAHs in sampling sites seemed to be related to different sources of PAHs. The large amount of low condensate ring structures in the sediments indicated oil pollution as the major source of PAHs pollution as it was found by Sericano et al., 2001 [40].

To assess the potential eco-risk of PAHs in Khuzestan coast sediments, the PAH concentrations which were already measured were compared with the effect-based sediment guideline values as suggested earlier in Long et al., 1995 [41] and Ningjing et al., 2010 [42] studies. The concentrations of B(a)A, F, AC, Ph and Ch at eight cases were higher than effect range-low (ERL) values, but they were lower than effect range-medium (ERM) values (Table 4). These results indicated that negative eco-risk are likely to occur, although with a likelihood below 50% effects in this area. However, other individual PAH concentrations were below ERL values which indicated a low risk of biological effect caused by PAHs (Table 4). Similar to this study, Imma Tolosa et al., 2005 [15] came to the same conclusions about the sediments in the southern part of the Persian Gulf (i.e., Bahrain, Qatar, UEA and Oman coast).

**Table 4:** Sediment PAHs concentration compared with ERL and ERM values

**PAHs**	**Guidelines**		**Sites**				
	**ERL**	**ERM**	**Arvand**	**Jafari**	**Zangi**	**Samayeli**	**Bahrakan**
B(a)A	261	1600	ERL < … < ERM	ERL < … < ERM	ERL < … < ERM	< ERL	< ERL
F	19	540	ERL < … < ERM	< ERL	< ERL	< ERL	< ERL
AC	44	640	ERL>	ERL < … < ERM	ERL < … < ERM	< ERL	nd
Ph	24	1500	< ERL	ERL < … < ERM	< ERL	< ERL	< ERL
Ch	384	2800	< ERL	ERL < … < ERM	< ERL	< ERL	< ERL
B(a)Py	430	1600	< ERL	< ERL	< ERL	nd	nd
Fl	600	5100	< ERL	< ERL	< ERL	< ERL	< ERL
Py	665	2600	< ERL	< ERL	< ERL	< ERL	< ERL
TPAHs	4022	44792	< ERL	< ERL	< ERL	< ERL	< ERL

nd: non detected.

The different patterns of PAHs found in the liver of mudskipper showed that there was large amount of the lower molecular weight pollution that is three-and four-ring structures. Low molecular weight PAHs found in the liver tissues might be the result of metabolic transformations of higher molecular weight PAHs occurring in fish liver [43]. Based on the findings of this re, It could be concluded that the PAH composition in fish liver was a sign of predominant derivation of PAH from oil pollution rather than from pyrolitic sources. A similar pattern was also found in the liver of *Epinephelus coioides* which exist in the southern part of the Persian Gulf [15].

Lower PAH concentrations in mudskipper liver tissues compare to the sediment might be the result of metabolic transformations of higher molecular weight PAHs occurring in fish liver [43,44]. Fish can convert up to 99% of the PAHs to metabolites within 24 hours of uptake, changing the pattern and the concentrations of PAHs in several tissues [32,45,46]. Moreover, half-life of PAHs is generally very short in all kinds of fish [43,47].

The Joint FAO/WHO Expert Committee on Food Additives has adopted a specification, which requires that the concentration of benzo[a]pyrene should not exceed a limit of 10 μg/kg [6,48-50]. In the current study, B(a)Py could be identified at detectable levels in the liver tissues of mudskipper from three sites (i.e., Arvand, Jafari and Zangi), while their values were much below the limit.

Eight and five PAH compounds were detected in water and gill samples, respectively, where two-and three-ring PAHs were dominated. This could be due to the fact that PAHs with lower solubility are attracted by particulate matters, depositing more in bottom sediments. Furthermore, PAHs are not concussively adsorbed by the gill systems of marine organisms although more soluble compounds have more persistency in seawater. They are more bioavailable and could be easily absorbed through gill membrane or food ingestion. Similar to this finding, Bummard,1998 [35] found that low molecular weight (LMW) compounds, rather than high molecular weight (HMW), are more abundant in marine organisms. The results of this study were in agreement with those of Law et al., 1997 [51] who reported high concentration of two- and three-ring PAHs in sea water around England. The concentrations of PAHs in sea water of Marmara Sea [52] were also comparable with the ones found in the present study.

There were wide variations in the total parent PAH concentration in the water, the sediment and the biota samples. The PAH compositions at each site were found to be different even when the discharges were almost similar. Obviously, each composition is a characteristic of the particular waste being discharged from. There was a river near the Arvand station introducing some terrestrial organic matters and waste discharge of the refineries which could therefore introduce high PAHs to the sediment of this site. Jafari, Zangi and Samayeli sample areas are located in Khoure-Mousa bay which is very shallow and stagnant having limited water circulation. Unfortunately, without receiving any pre-treatments, the local industries, the refineries and the ports discharge their wastes to this bay. Regarding PAH concentration resulted from the pollutions, in the sample areas, Jafari showed higher total parent PAH concentration. Presence of Mahshahr Special economic and petroleum zone as a major refineries zone in North Western part of Persian Gulf which introduce high PAHs to Jafari site may be responsible for the higher contamination level observed at this station.

The Hepatosomatic index (HSI) values showed that there were significant differences among the sampling stations. However, no significant differences were observed among the sites in case of condition factors (CFs). A high correlation was found between HSI and total parent PAH concentrations in the liver. This indeed indicates that accumulation of PAHs in mudskipper can influence its liver tissue as a target organ of the fish to detoxify PAH. HSI are potentially indicative of toxicant effects. They provide useful information about energy reserves and the ability of individuals to tolerate chemical pollution challenge or other kinds of environmental stress [53-56].

In order to clarify potential sources or origins of PAHs in marine sediments, several PAH rations have been recommended [12,57,58]. The Fl/Py ratios of the sediment and the liver tissues pointed to petrogenic origin for Arvand, Zangi, Samayeli, and Bahrakan, Nonetheless, pyrolytic source was established at Jafari site. In case of the water and the gill samples, the values of Fl/Py ratios strongly implied a pyrolytic origin except for Bahrakan site. Although Ch/B(a)A ratio suggested a petrogenic origin for the sediment and the liver tissues at all sampling sites, it indicated a pyrolytic origin for water at Jafari and Arvand and petrogenic source at Zangi. In addition, PAH fingerprints in all tissues together with the adjacent sediments and water showed an overall predominance of LMW PAHs. The high presence of low condensate ring structures in all samples indicated oil pollution as the major source of PAHs contamination. Most importantly, different origins of PAHs were identified by the diagnostic ratios used in this study. This finding might be owing to the complexity of the parameters which determine PAHs distributions in the environment.

## Conclusion

This study provides important information on PAH concentrations in surface sediments, water and *B. dussumieri* from north western part of the Persian Gulf. The Persian Gulf is generally considered as an extremely polluted ecosystem with respect to oil and refineries. This is particularly true about Khuzestan coast which is located in the north west of the Persian Gulf. This study, however, showed that PAH concentrations in this region did not exceed the NOAA sediment quality guideline value for the effects range low (ERL). Our results also revealed a negative eco-risk effects occasionally occur in this area. The higher presence of low condensate ring structures reflected a predominant origin of petrogenic and some cases of pyrolitic sources in North West of Persian Gulf. These findings suggest that mudskipper *(B. dussumieri*) is not robust as a bioindicator of PAHs pollution in marine ecosystems, further research is needed. Regular monitoring of the Persian Gulf area is suggested in order to determine if any dumping activities have occurred in this particular area.

## Competing interests

We have received funding from national institute of oceanography that has applied for PAHs analysis relating to the content of the manuscript.

## Authors’ contributions

AM: participated in the design of the study and performed the statistical analysis. Both authors read and approved the final manuscript.
